# Expression of DNAJB12 or DNAJB14 Causes Coordinate Invasion of the Nucleus by Membranes Associated with a Novel Nuclear Pore Structure

**DOI:** 10.1371/journal.pone.0094322

**Published:** 2014-04-14

**Authors:** Edward C. Goodwin, Nasim Motamedi, Alex Lipovsky, Rubén Fernández-Busnadiego, Daniel DiMaio

**Affiliations:** 1 Department of Genetics, Yale School of Medicine, New Haven, Connecticut, United States of America; 2 Department of Cell Biology, Yale School of Medicine, New Haven, Connecticut, United States of America; 3 Department of Therapeutic Radiology, Yale School of Medicine, New Haven, Connecticut, United States of America; 4 Department of Molecular Biophysics & Biochemistry, Yale School of Medicine, New Haven, Connecticut, United States of America; 5 Yale Cancer Center, New Haven, Connecticut, United States of America; University of Toronto, Canada

## Abstract

DNAJB12 and DNAJB14 are transmembrane proteins in the endoplasmic reticulum (ER) that serve as co-chaperones for Hsc70/Hsp70 heat shock proteins. We demonstrate that over-expression of DNAJB12 or DNAJB14 causes the formation of elaborate membranous structures within cell nuclei, which we designate DJANGOS for *D*NA*J*-*a*ssociated *n*uclear *g*l*o*bular *s*tructures. DJANGOS contain DNAJB12, DNAJB14, Hsc70 and markers of the ER lumen and ER and nuclear membranes. Strikingly, they are evenly distributed underneath the nuclear envelope and are of uniform size in any one nucleus. DJANGOS are composed primarily of single-walled membrane tubes and sheets that connect to the nuclear envelope via a unique configuration of membranes, in which the nuclear pore complex appears anchored exclusively to the outer nuclear membrane, allowing both the inner and outer nuclear membranes to flow past the circumference of the nuclear pore complex into the nucleus. DJANGOS break down rapidly during cell division and reform synchronously in the daughter cell nuclei, demonstrating that they are dynamic structures that undergo coordinate formation and dissolution. Genetic studies showed that the chaperone activity of DNAJ/Hsc70 is required for the formation of DJANGOS. Further analysis of these structures will provide insight into nuclear pore formation and function, activities of molecular chaperones, and mechanisms that maintain membrane identity.

## Introduction

A defining characteristic of eukaryotic cells is the complex architecture of their membrane structures. The plasma membrane isolates the cell from the extracellular environment, and the membranes of organelles organize biochemical reactions at specific intracellular locations. These membranes also harbor specific proteins that carry out specialized functions including enzymatic reactions and various pore and channel activities. Normal cell function requires that membranes maintain their discrete identities and dedicated components. This poses a particular challenge for the nuclear membranes of metazoans, which disappear during mitosis and reform in the daughter cells following cytokinesis [1], [2]. Therefore, processes must exist to ensure the coordinated breakdown and formation of the nuclear envelope during the cell cycle and the rapid reestablishment of membranes containing their proper constituents.

The nuclear envelope consists of the outer nuclear membrane (ONM), the inner nuclear membrane (INM), and the shared intermembrane space, which is continuous with the lumen of the endoplasmic reticulum (ER) [1], [3]. A dense lamin mesh composed of lamin A and lamin B underlies the INM. The cytoplasm and nucleus are connected by pores that contain massive protein assemblies called nuclear pore complexes (NPCs), which are located at the junction of the INM and ONM [4]. NPCs mediate the exchange of molecules between the cytoplasm and nucleus by imposing a diffusion barrier to molecules >30 kD and by promoting the active transport of macromolecules bound to nuclear transport receptors [5], [6]. Thus, the nuclear membranes and their embedded NPCs partition the nuclear volume from the cytoplasm and its contents, allowing spatial separation of important cellular processes like DNA replication and translation.

The NPC is composed of ∼30 distinct proteins termed nucleoporins/nups, which form modular building blocks that construct the transmembrane, inner ring, and outer ring complexes that form the scaffold of the NPC, which surrounds a ∼40 nm diameter central transport channel [4], [7]–[9]. A single continuous membrane sheet is separated into ONM and INM domains by a highly curved membrane at the periphery of the NPC [6], [7]. Transmembrane protein components of the NPC impede the movement of macromolecules past the NPC, thus restricting various proteins to the ONM or the INM. The ONM is continuous with the membrane of the endoplasmic reticulum (ER) and shares membrane components with the ER. Integral INM proteins such as emerin and lamin B receptor (LBR) are inserted into the ER membrane during synthesis and must move past the NPC to reach the INM, whereas other ONM proteins do not transit past the NPC [10]. Furthermore, it is thought that the ER “stores” nuclear envelope components during mitosis, yet NPCs quickly reassemble after cell division, and INM and ONM components segregate to their correct locations [1], [2], [11].

Membranes can also tunnel into the nucleus. An invagination of both the INM and ONM, named the type II nucleoplasmic reticulum, retains the normal topology between the ONM, the INM, and the underlying lamina, and may contain cytoplasmic material within the tube formed by the invaginations [12]. Occasionally, a single-walled membrane protrudes from this structure or the INM and extends farther into the nucleus to form the type I nucleoplasmic reticulum [12]. Although the function of the nucleoplasmic reticulum is unknown, deep penetration into the nucleus may allow localized signaling events internal to the nuclear periphery. In addition, the nucleoplasmic reticulum may allow directional transport of adeno-associated virus within the nucleus [13].

We recently reported that two closely related ER proteins, DNAJB12 (designated B12) and DNAJB14 (B14), are essential for infection by the DNA tumor virus, simian virus 40 (SV40) [14]. Knock-down of either protein reduced SV40 infectivity by approximately 50-fold by preventing the exit of the disassembling viral capsids from the ER lumen prior to nuclear entry. DNAJ proteins contain a highly conserved J-domain and usually act as co-chaperones by increasing the folding activity of catalytically active Hsp/Hsc70 chaperones [15], [16]. B12 and B14 are type II transmembrane proteins, which restricts the J-domain to the cytoplasmic face of the ER membrane. In association with Hsc70, they are involved in endoplasmic reticulum-associated degradation (ERAD), an ER quality control mechanism [17]–[19] in which improperly folded proteins in the ER lumen or membrane are extracted from the ER and degraded in the cytoplasm [20].

We report here that overexpression of B12 or B14 causes cells to form elaborate intranuclear membranous structures that stain intensively for B12, B14, and Hsc70. These structures were generally of uniform size within a nucleus, formed coordinately in individual nuclei, and disappeared synchronously during cell division. Genetic experiments revealed that a functional J-domain and Hsc70 were required for the formation of these structures, which appear to emerge from a novel configuration of membranes in proximity to atypical NPCs. These experiments revealed a novel consequence of DNAJ/Hsc70 activity, namely dramatic remodeling of membranes associated with a subset of nuclear pores, which results in the coordinate influx of membranes into the nucleus.

## Results

### Over-expression of DNAJB12 or DNAJB14 causes the formation of nuclear structures

**Figure 1:**
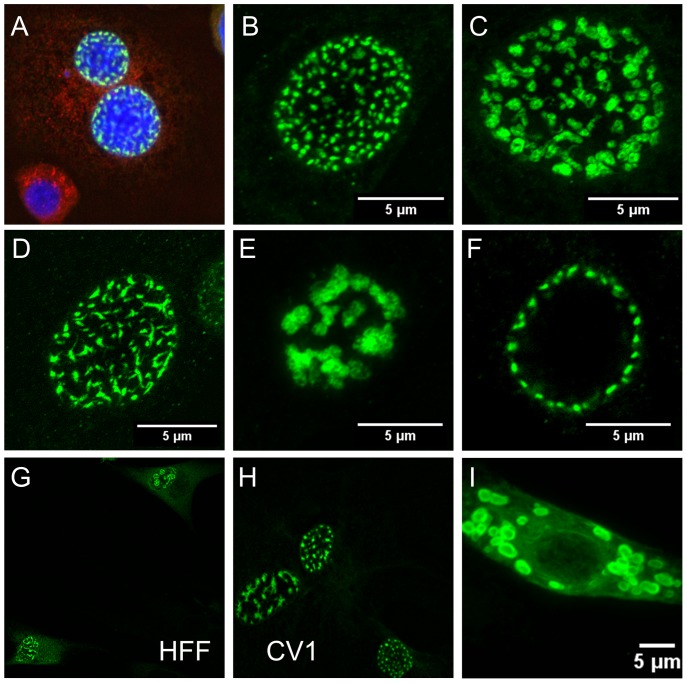
DNAJB12 induces formation of DJANGOS. Immunofluorescent staining of DJANGOS in cells over-expressing HA-tagged DNAJB12 (A–F) or DNAJB12 fused to tGFP (G–I), detected with anti-HA or anti-tGFP antibodies (both green), respectively. All images except (F) are from confocal stacks compressed along the Z-axis to create a single image. **A.** HeLa cells were also stained with anti-PDI (red) and DAPI (blue, to visualize nuclei). **B–E.** HeLa cell nuclei displaying different varieties of DJANGOS. **F.** Single confocal slice midway up the nucleus showing regular distribution of DJANGOS under the nuclear envelope in a HeLa cell. **G and H.** Nuclei of human foreskin fibroblasts and CV1 monkey cells, respectively. **I.** Rare cytoplasmic form of DJANGOS, seen here at lower magnification in a HeLa cell lacking the more common nuclear forms.

**Figure 2:**
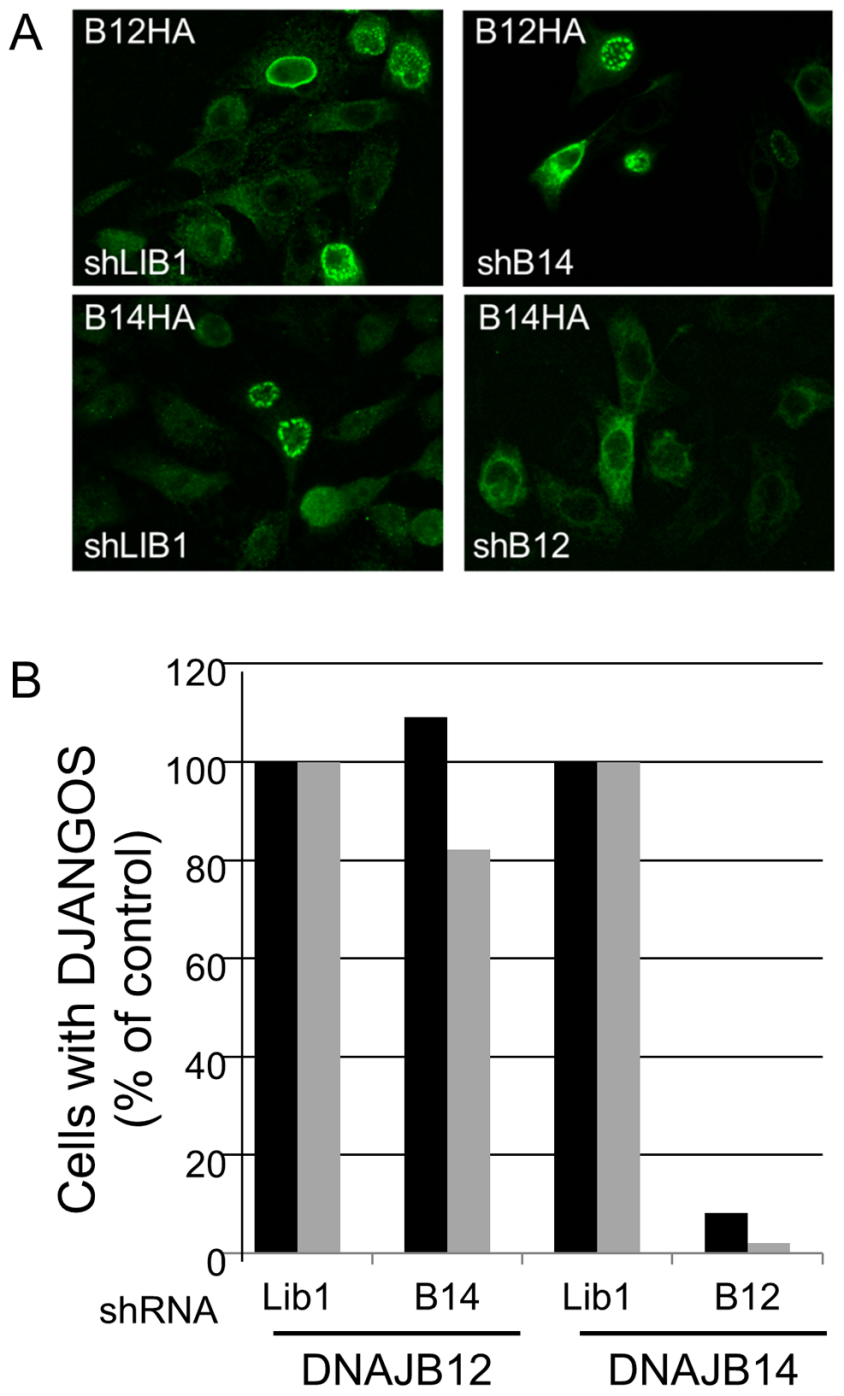
Role of B12 and B14 in formation of DJANGOs. **A.** Images are individual confocal slices of HeLa cells stained with anti-HA to detect HA-tagged B12 or B14. The left panels show cells expressing an shRNA targeting an irrelevant gene (shLib1), the right panels show cells expressing an shRNA targeting B14 (top) or B12 (bottom). The top panels show cells over-expressing B12-HA while the bottom panels show cells over-expressing B14-HA. **B.** Random microscopic fields of cells treated as in panel A were scored for the presence of DJANGOS in two independent experiments (shown in black and grey bars) and normalized to the values for cells expressing Lib1 control shRNA. In these experiments, B12 induced DJANGOS in approximately 29% of control cells, and B14 induced DJANGOS in approximately 11% of control cells. The data were subjected to an F-test followed by an unequal variance t-test. B12 knock-down significantly reduced the ability of B14 to induce DJANGOS as compared to cells expressing the control shRNA (p<0.021), whereas B14 knockdown did not affect the ability of B12 to induce DJANGOS (p <0.8).

In the course of investigating the requirement for the cellular DNAJB12 and DNAJB14 proteins (B12 and B14, respectively) for infection by SV40, we examined HeLa cells that expressed HA-tagged B12. Although most cells displayed a reticular ER-like HA-staining pattern, a fraction of cells also displayed intense punctate nuclear HA staining (Figure 1A). To explore this observation in more detail, concentrated retroviral stocks were used to generate HeLa cells, CV1 cells and human primary foreskin fibroblasts (HFFs) over-expressing HA-tagged B12 or B14, which were then subjected to indirect immunofluorescence for HA and visualized by confocal microscopy. Five to thirty five percent of the cell nuclei in different experiments display numerous punctate structures, which stain intensely with the HA antibody (Figure 1). Expression of either B12 or B14 induces these nuclear structures (Figure S1A), although B12 is more active (Fig. 2B). B12 or B14 staining in the ER or nuclear envelope is much fainter than these intensely staining nuclear structures. Only faint background nuclear staining is observed in control cells not expressing tagged B12 or B14 (Figure 1A).

The most common staining pattern induced by B12 or B14 in HeLa cells is shown in Figure 1B, where ∼150 spots of HA reactivity appear evenly distributed in the nucleus. Nuclei containing larger and less numerous spots with a heterogeneous internal structure are also observed (Figure 1C), as are nuclei containing structures with a “wispy” appearance (Figure 1D). Rare nuclei contain a smaller number of huge pleomorphic structures (Figure 1E). Strikingly, the structures within a given nucleus display similar sizes and shapes. Examination of single confocal slices revealed that the structures are not distributed throughout the nuclear volume, but rather arrayed in a regular pattern underneath the entire circumference of the nuclear envelope (Figure 1F). These structures are also observed in CV1 cells and HFFs that over-express B12 or B14 (Figures 1G and 1H; data not shown). We name these nuclear structures *D*NA*J*-*a*ssociated *n*uclear *g*l*o*bular *s*tructures, DJANGOS. In rare cells over-expressing B12, the HA antibody stains large, oval cytoplasmic structures (Figure 1I). These cytoplasmic structures were found in cells with or without nuclear DJANGOS.

DJANGOS are observed in HeLa cells if the over-expressed B12 contains the HA tag at either terminus, or if turboGFP (tGFP) or mCherry is fused to its carboxy-terminus (Figure S1B, left panel; Figure S2; data not shown). When tGFP-tagged B12 was co-expressed with HA-tagged B14, both proteins co-localize in individual DJANGOS (Figure S1B). We also developed a monoclonal antibody that recognizes untagged, endogenous B12 protein (Figure S1C). This antibody generates faint ER-type staining in control cells expressing only endogenous B12 (data not shown), but it stains DJANGOS indistinguishable from those described above in HeLa and CV1 cells over-expressing tagged or untagged B12 (Figure S1D, data not shown). Thus, DJANGOS formation does not require the addition of epitope tags or fusion to other proteins.

To examine in more detail the requirements for DJANGOS formation, we infected HeLa cells with retroviruses expressing a control shRNA or an shRNA directed against B12 or B14, and stable cell lines with reduced B12 or B14 expression were established. Knock-down and control cells were then infected with retroviruses expressing HA-tagged B14 or B12, and DJANGOS formation was assessed. As expected, B12 or B14 over-expression induces DJANGOS in cells expressing the control shRNA (Figure 2A, left panels). Over-expression of B12 in the B14 knock-down cells also induces DJANGOS (Figure 2A, upper right), even though B14 expression remains repressed (data not shown), demonstrating that B14 is not required for formation of DJANGOS by B12. In contrast, B14 over-expression does not induce the formation of DJANGOS in the B12 knock-down cells (Figure 2A, lower right), showing that B12 expression is required for B14 to induce DJANGOS. These results are quantified in Figure 2B. Because B14 over-expression does not up-regulate B12 (data not shown), this result also suggests that endogenous levels of B12 are sufficient for DJANGOS induction by B14.

### DJANGOS contain Hsc70, ER and nuclear envelope markers

**Figure 3:**
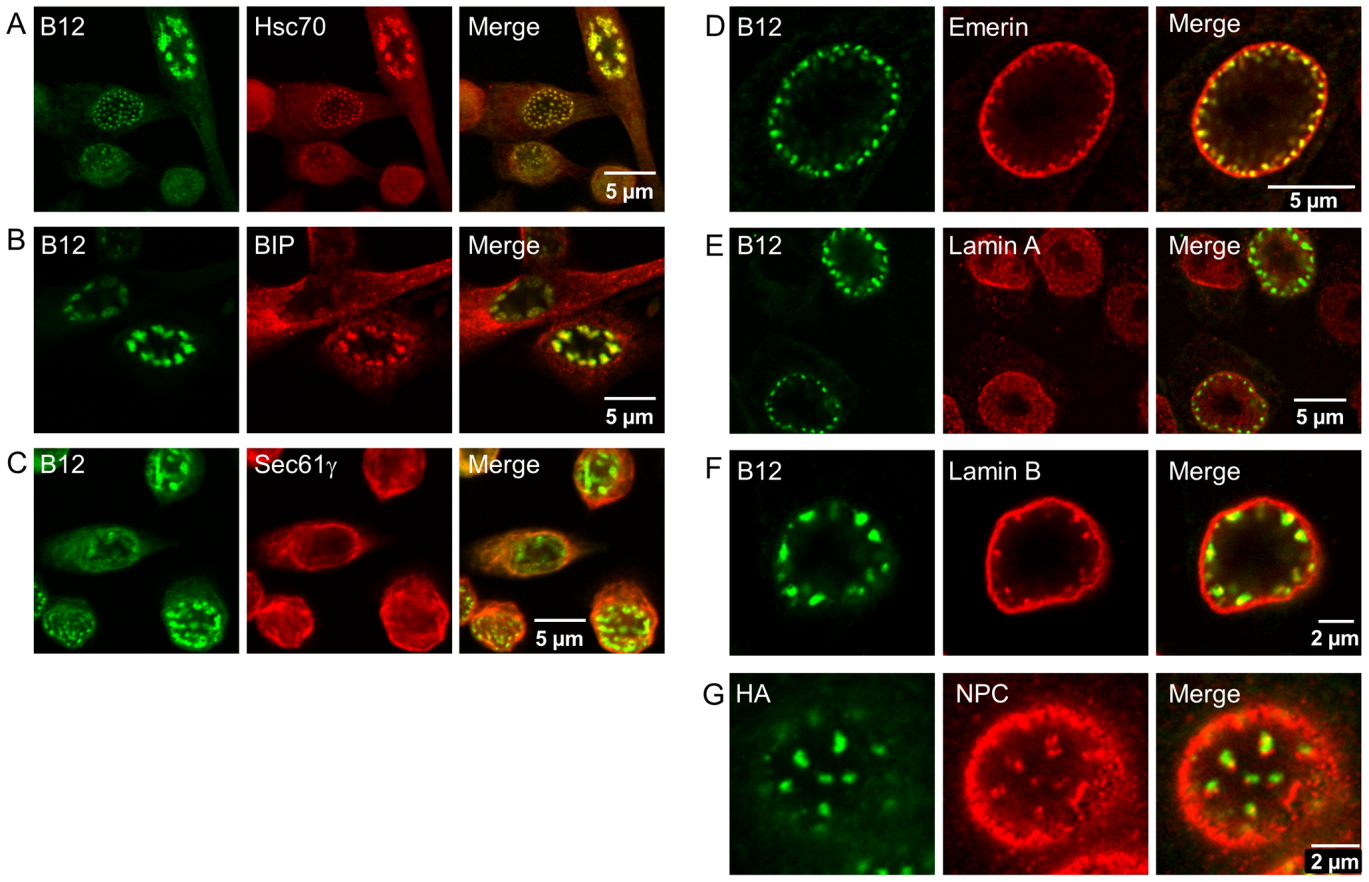
Co-localization of B12 with Hsc70 and other proteins in DJANGOS. HeLa cells over-expressing B12-HA were stained with anti-B12 (A–F) or anti-HA (G) to detect B12 (in green) and with antibodies specific for the indicated cellular protein (in red). Overlapping signals in the merged images on the right are shown in yellow. The same confocal slice is shown in each row of panels. **A.** Hsc70. **B.** BiP. **C.** Sec61γ. **D.** Emerin. This section is from the same nucleus shown in Figure 1B. **E.** Lamin A. **F.** Lamin B. **G.** NPC. This slice is near the bottom of the nucleus.

We used immunofluorescence to determine if B12 and B14 co-localized with their putative Hsc70 partner and ER or nuclear markers in DJANGOS. None of the antibodies we used stained DJANGOS-like structures in the absence of B12 or B14 over-expression (data not shown). An antibody recognizing cytoplasmic Hsc70 shows strong staining of DJANGOS and co-localization with the B12 signal (Figure 3A). The luminal ER proteins BiP and protein disulfide isomerase (PDI) and the transmembrane ER protein calnexin also co-localize with B12 and B14 in DJANGOS (Figure 3B, and data not shown). In contrast, a marker of the rough ER membrane, Sec61γ, does not co-localize with DJANGOS (Figure 3C). We also stained for the ER membrane-resident reticulons (RTNs), which are involved in membrane bending. The antibody recognizing both RTN1 and RTN2 stains DJANGOS, whereas antibodies to RTN2, RTN3 or RTN4 do not (Figure S2, and data not shown). DJANGOS were also strongly stained with an antibody to emerin (Figure 3D), an INM protein [21]. The nuclear membrane markers, lamin B, lamin B receptor (LBR), but not lamin A, are present in the DJANGOS (Figures 3E and 3F and Figure S2). Lamin B staining is typically restricted to just an edge of the larger DJANGOS. In cells over-expressing B12,we see nuclear pore complex (NPC) staining at the nuclear margin and at the periphery of the larger DJANGOS (Figure 3G), suggesting that NPCs, like lamin B, are restricted to only a portion of these structures. The pattern of staining described above indicates that DJANGOS are intrusions of the ER into the nuclear volume, containing components of nuclear and ER membranes, as well as the chaperone Hsc70.

In HeLa, CV1, and HFF cells lacking B12 or B14 over-expression, staining for emerin revealed simple, small intranuclear structures that appear to correspond to the nucleoplasmic reticulum (Figure S2, bottom panels; data not shown). These structures are also stained with antibodies recognizing lamin B, NPC and faintly with antibodies to endogenous B12 (Figure S2; data not shown). However, these structures were much smaller, less numerous, and stained much more faintly than DJANGOS.

### Ultrastructure of DJANGOS

**Figure 4:**
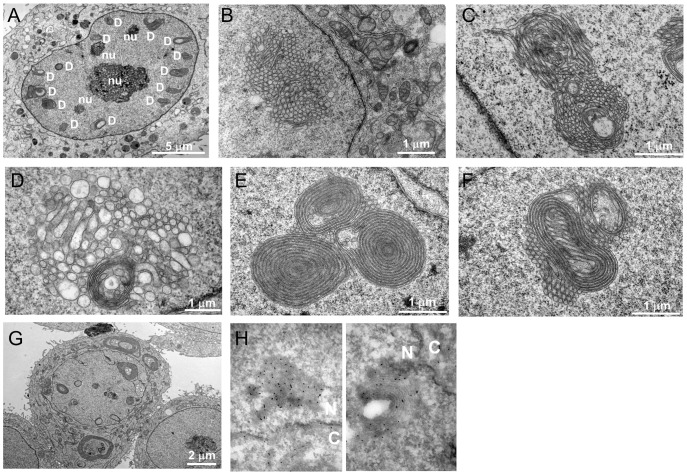
Ultrastructure of DJANGOS in HeLa cells. **A.** A low power electron micrograph of a cell over-expressing B12-HA shows multiple DJANGOS, labeled “D,” regularly spaced underneath the nuclear envelope. The dark granular objects labeled “nu” are nucleoli. **B.** Image of a cell over-expressing B12-HA and E2 shows intranuclear, tightly clustered, single-walled membrane tubes. **C.** Worm-like membrane tubes seem to transition from the cross-sectioned tubes in DJANGOS from cells over-expressing B12-HA. **D.** Cells expressing B14-HA and E2 also show intranuclear DJANGOS. The nuclear envelope and cytoplasm are visible in the lower right. **E.** Cells expressing B12-tGFP and E2 display concentric multi-lamellar nuclear whorls in close apposition to simple tubes. **F.** Complex membranous structure in cells expressing B12-tGFP and E2. **G.** Cytoplasmic and nuclear concentric multi-lamellar whorls in cells expressing B12-tGFP and E2. **H.** High-power EM images of cells over-expressing B12-HA stained with anti-BiP antibody and gold bead-conjugated secondary antibody. Membranes appear as a negative image, and the black spots are gold beads indicating sites of BiP reactivity. “N” and “C” indicate nucleus and cytoplasm, respectively.

We used transmission electron microscopy (EM) to visualize DJANGOS. Up to 20% of HeLa cells over-expressing B12 or B14 contain complex intranuclear membranous structures, which are absent from control cells. These structures tend to be near the nuclear periphery, and the sizes of these structures in individual nuclei are in general quite uniform (Figure 4A). To increase the likelihood of finding DJANGOS by EM, we exploited the observation that their frequency is increased several-fold in HeLa cells by expression of the bovine papillomavirus E2 protein (data not shown). E2 induces B12 and B14 expression and represses the endogenous HPV18 oncogenes in these cells [22], leading to growth arrest. There are no apparent differences between DJANGOS formed in the presence or absence of E2.

In most cases, DJANGOS consist primarily of clusters of small circular structures, which appear to be assemblies of dozens of tightly-associated tubes seen in cross-section (Figure 4B), although smaller clusters of only a few tubes are also observed (Figure S3A). These structures are single-walled (*i.e.*, appear to consist of a single lipid bilayer), of a similar diameter within a given cluster (ranging from approximately 70 to120 nm), and devoid of the electron-dense lamina. We also observed worm-like pairs of single-walled membranes that in some cases are next to the circular structures or seemed to extend from them (Figure 4C), suggesting that the circular and tube-like appearance of these structures reflect their different orientations relative to the EM section. Similar structures are induced by B14 (Figure 4D). Membrane sheets wrapped into large, concentric multi-lamellar whorls adjacent to simple tubes are also present (Figure 4E). In some complex DJANGOS, intertwined tubes transition from the circular to worm-like forms and are wrapped by other tubes or membrane sheets (Figure 4F). In rare cells, concentric multi-lamellar whorls are present in the cytoplasm (Figure 4G). To confirm that these structures correspond to those seen by immunofluorescence, we performed immunoEM with antibody recognizing BiP. Immunogold staining showed specific signal within the tubular and concentric membranous structures in the nucleus of cells expressing B12-HA as well as the expected staining of the nuclear surface and ER (Figure 4H). We conclude that the membranous structures seen by EM and the DJANGOS observed by immunofluorescence are the same structures.

In addition to these complex structures, membrane-lined, simple invaginations of the nuclear membranes into the nucleus were found in approximately 20% of HeLa cells, whether or not they over-expressed B12 or B14 (Figures S3B and S3C). These structures, which are bound by double membranes, show lamin density similar to that underlying the INM and occasionally contained classic NPCs. Thus, they appear to represent the type II nucleoplasmic reticulum. Usually no more than one or two of these structures were observed in the entire nuclear section, and they never comprised small tubes and whorls as described above in cells over-expressing B12 or B14. Therefore, they are distinct from the elaborate nuclear structures induced by B12 and B14.

### DJANGOS emerge from double-membrane bodies connected to the nuclear margin via atypical nuclear pores

**Figure 5:**
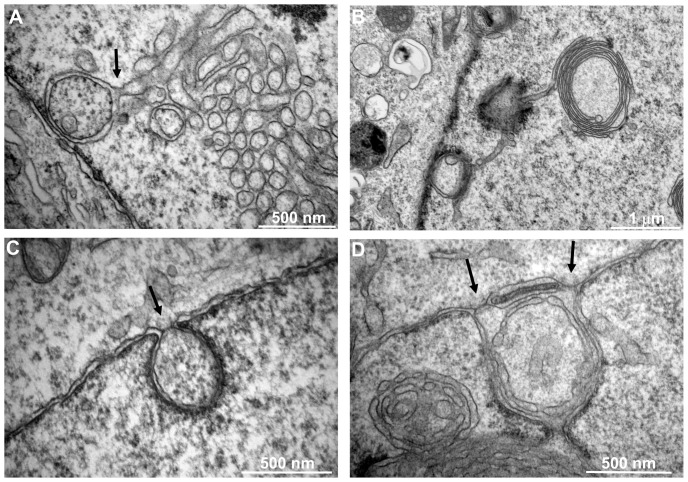
DJANGOS emerge from atypical nuclear pores. HeLa cells over-expressing B12-HA were visualized by electron microscopy. **A.** A double-walled membrane body within the nucleus connects to a tube (arrow), which then intermingles with other tubes in various orientations. The cytoplasm is at the lower left. **B.** Double-membrane body in proximity to the nuclear envelope connects to a complex structure extending into the nucleus. Cytoplasm is at left. **C.** Simple double-membrane structure extending into the nucleus from an atypical nuclear pore (indicated by the arrow). Cytoplasm is at top. **D.** Complex double-membrane structure containing two atypical nuclear pores (indicated by the arrows) connected to tubular structures inside the nucleus. Cytoplasm is at top.

The electron micrographs provided clues regarding the biogenesis of DJANGOS. In addition to single-membrane structures, we occasionally observed double-membrane oval bodies close to the nuclear margin. In several cases, these bodies connect to a single-walled tube, which in turn connects to a larger cluster of tubes (Figure 5A) or an incipient concentric nuclear whorl (Figure 5B). These images suggest that the tubular and concentric structures are derived from these double-membrane bodies.

In examining approximately 500 cells with DJANGOS, we observed 16 direct connections of DJANGOS to the nuclear envelope, all of which involve double-membrane bodies continuous with both the INM and ONM. These bodies displayed a novel nuclear pore structure, in which the gray-staining NPC is not anchored in its usual position at the junction of the INM and ONM but rather associates exclusively with the ONM (Figures 5C and 5D). Some of these bodies appear as relatively simple structures (Figure 5C), in which the double-layered nuclear envelope invaginates into the nucleus at an atypical nuclear pore while maintaining continuous association with dark staining lamin. A related but more complex structure contains two nearby atypical nuclear pores and membranes in the same configuration as described above, connected to membranes within the nucleus (Figure 5D). However, we note that the majority of NPCs in cells containing DJANGOS appear normal.

**Figure 6:**
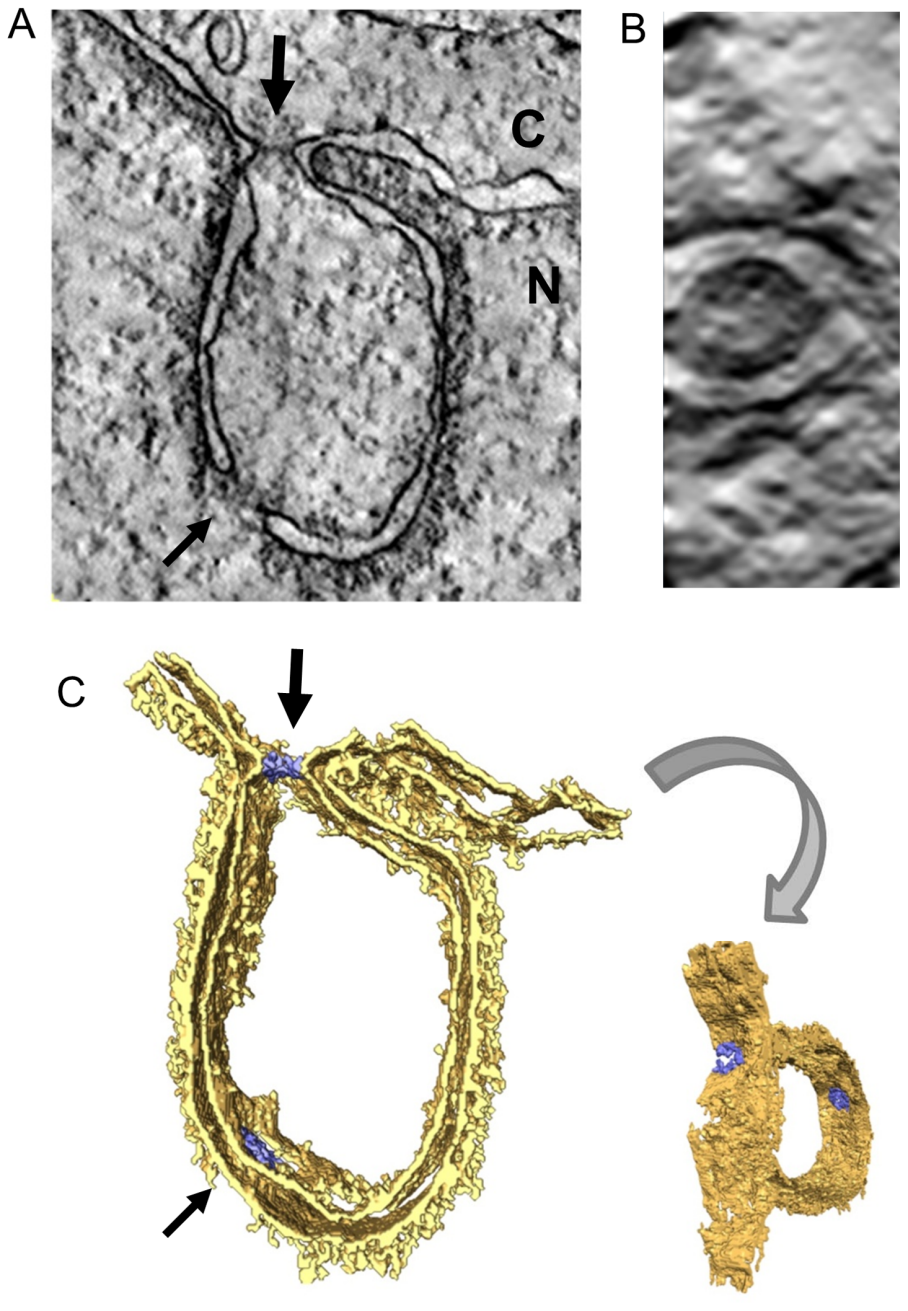
Electron tomography of DJANGOS. **A.** Tomographic slice of an EM thick section showing a nuclear pore-associated structure formed in HeLa cells expressing B12-HA. Cytoplasm and nucleus are labeled with “C” and “N,” respectively. The thick arrow points to an atypical nuclear pore structure in cross-section at the junction with the nuclear envelope; the thin arrow points to a classic nuclear pore in the double membrane inside the nucleus. **B.** End-on view of the atypical nuclear pore at the neck of the double-membrane structure. **C.** 3D rendering of the tomogram shows the NPCs in blue. Also see Movie S1.

To further explore this configuration of membranes at atypical nuclear pores, thick EM sections were subjected to tomographic analysis. We captured one double-membrane body with a direct connection to the nuclear envelope. A static image from midway through the section shows the same type of structure as in Figure 5C (Figure 6A), and an end-on view of the atypical pore at the top revealed an intact circular structure (Figure 6B). A 3D rendering showed that this double-walled structure emerges from the entire circumference of the NPC, which appears to allow the membranes to flow around it into the nuclear interior, like the neck of a balloon (Figure 6C). This structure also contains a normal-appearing NPC distal to the nuclear margin. Movie S1 shows the complete stack and animated 3D rendering.

### DJANGOS form rapidly and synchronously

The elaborate structure of DJANGOS suggested that they undergo a complex biogenesis program. We used live-cell imaging to explore the dynamics of DJANGOS formation. HeLa, HeLaM, or CV1 cells were transfected with plasmids expressing B12 fused to the fluorescent protein mCherry (B12-mCherry) and LBR fused to enhanced green fluorescent protein (LBR-eGFP). After 36 hours, the cells were subjected to live-cell imaging, with confocal fluorescence images acquired at fifteen minute intervals. Expression of LBR-eGFP alone does not induce DJANGOS formation (data not shown), but co-transfection of B12-mCherry and LBR-eGFP caused the formation of DJANGOS containing B12 and LBR in all three cell types. Nuclei undergo a transition from being devoid of structures to having numerous small DJANGOS near the nuclear margin within a few frames (*i.e.*, in less than an hour); once DJANGOS appear, they grow in size over a period of two to four hours (Movie S2).

**Figure 7:**
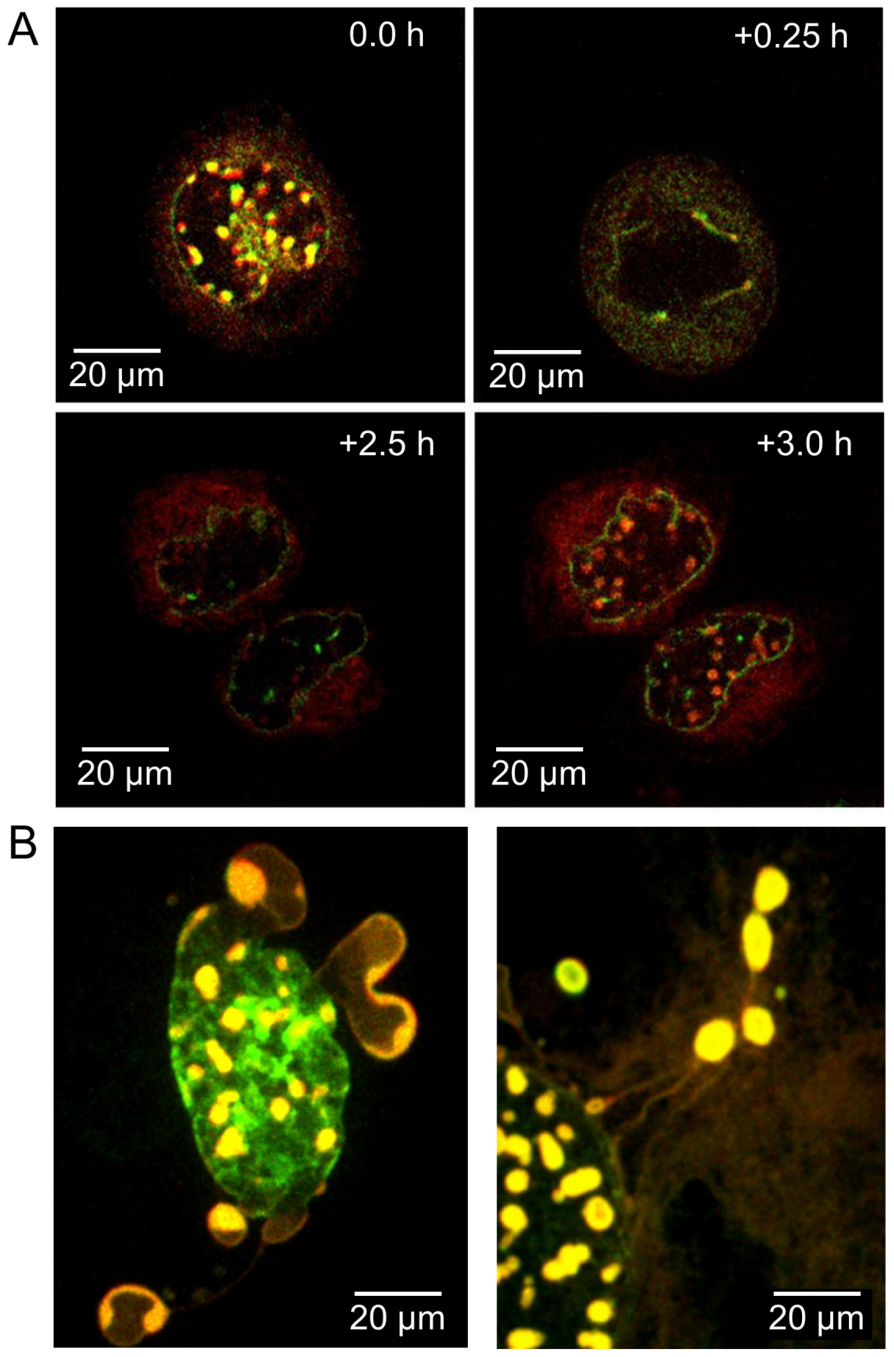
Live-cell imaging of DJANGOS. Static images retrieved from live cell imaging of HeLaM or CV1 cells co-transfected with plasmids expressing B12-mCherry and LBR-eGFP. **A.** Top panels show two images of a HeLaM cell separated by 15 minutes acquired shortly before mitosis. Bottom panels shows images acquired one hour apart of the daughter cells of the cell shown in the top panels. See Movie S3. **B.** Left panel shows cytoplasmic billowing of B12 structures in CV1 cells; right panel shows cytoplasmic B12 “balloons on strings” in CV1 cells. See Movies S4 and S5.

Some HeLaM cells containing DJANGOS undergo cell division during the time of observation, demonstrating that DJANGOS do not prevent cell cycle progression. Strikingly, prior to nuclear envelope breakdown, all DJANGOS in the cell disappear within a single frame (*i.e.*, within 15 minutes), and after the completion of division they synchronously reform in both daughter nuclei (Figure 7A; Movie S3). The formation of DJANGOS in interphase cells as well as in cells that had recently undergone cell division indicated that their appearance is not strictly cell-cycle dependent. In addition, the synchronous appearance of multiple structures in daughter cells that recently completed division suggested coordinated formation of DJANGOS at numerous sites in the nucleus rather than independent, local growth.

In some cells, we observed dynamic heterogeneous protrusions of structures containing B12 and LBR into the cytoplasm. Some of these structures are pleomorphic, dynamic blebs, which “billow” out from the nucleus and occasionally later partially retract into it (Figure 7B, left panel; Movie S4). We also observed balloon-like structures that emerge from the nucleus and extend into the cytoplasm while remaining tethered to the nucleus by long string-like structures (Figure 7B, right panel; Movie S5). Cells remain viable despite the presence of these structures.

### Formation of DJANGOS requires DNAJ/Hsc70 chaperone activity

**Figure 8:**
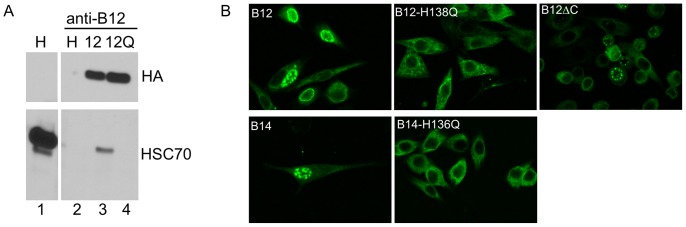
DJANGOS formation requires DNAJ/Hsc70 activity. **A.** RIPA buffer extracts were prepared from unmodified HeLa cells (H) or cells transduced with genes encoding wild-type B12-HA (B12) or the H138Q-HA mutant (12Q). Extracts were immunoprecipitated with anti-B12 and subjected to SDS-polyacrylamide gel electrophoresis, or electrophoresed without immunoprecipitation (lane 1). Top and bottom panels show anti-HA and Hsc70 immunoblots, respectively. **B.** HeLa cells were infected with retroviruses expressing the indicated wild-type or mutant B12-HA (top panels) or B14-HA (bottom panels) gene and stained with anti-HA to detect B12 or B14. Images are individual confocal slices. The left panels show cells expressing wild-type B12 or B14, the middle panels the J-domain mutants and the right panel the carboxy- truncated B12ΔC.

DNAJ proteins contain a highly conserved J-domain that stimulates the ATPase activity of their associated Hsp70/Hsc70 chaperone partners. As noted above, Hsc70 co-localizes with B12 in DJANGOS (Fig 3A). To determine whether the DNAJ co-chaperone activity of B12 and B14 is required for the formation of DJANGOS, we constructed a histidine to glutamine mutation in the essential HPD motif [23] of HA-tagged B12 and B14 (H138Q and H136Q respectively) and tested the ability of the mutants to associate with Hsc70 and to induce the appearance of DJANGOS. We prepared RIPA extracts from HeLa cells expressing wild-type or mutant HA-tagged B12, immunoprecipitated with the anti-B12 monoclonal antibody, and immunoblotted with anti-HA to examine expression of B12. As shown in Figure 8A, top panels, similar levels of wild-type and mutant B12 are expressed in the cells (lanes 3 and 4). We also immunoblotted the anti-B12 immunoprecipitates with an Hsc70 antibody. As shown in Figure 8A, bottom panels, the B12 antibody co-immunoprecipitates a small percentage of Hsc70 from cells expressing wild-type HA-tagged B12 but not from cells expressing only endogenous B12 (lanes 2 and 3), demonstrating that over-expression is required to detect the B12/Hsc70 interaction. Hsc70 is not co-immunoprecipitated from cells over-expressing the B12 J-domain mutant (lane 4). These results demonstrated that when B12 is over-expressed, it stably associates with its Hsc70 chaperone partner in a manner that requires a functional J-domain.

The wild-type and mutant forms of B12 and B14 display similar ER localization in most cells (Figure 8B, left and center panels). In the experiment shown in Fig. 8B, wild-type B12 and B14 induce the formation of DJANGOS in 31% and 7.4% of the cells, respectively. In contrast, we did not observe a single nucleus with DJANGOS in >1000 cells expressing either J-domain mutant. Similar results were obtained in three independent experiments, demonstrating that DNAJ co-chaperone function is required for the formation of DJANGOS. We next used RNA interference to test if Hsc70 is also required for DJANGOS formation. HeLa cells expressing B12-HA were transfected with an siRNA targeting GFP or with siRNAs targeting Hsc70 and assayed three days later for the presence of DJANGOS by immunofluorescence. As shown in Table 1, knock-down of Hsc70 causes a statistically significant 2 to 4-fold reduction in the fraction of cells with DJANGOS in two independent experiments. We obtained similar results when the Hsc70 siRNAs are combined. Taken together, these results show that DNAJ/Hsp70 chaperone activity is required for efficient DJANGOS formation.

**Table 1:** Knockdown of Hsc70 reduces B12-HA DJANGOS.

	siRNA	Positive/Total Cells	% GFP	p-value
**Experiment 1**	GFP	185/1115		
	Hsc70 pool	68/872	47	3.3×10^−4^
**Experiment 2**	GFP	149/1223		
	Hsc70 #1	36/798	37	5.7×10^−4^
	Hsc70 #2	86/1326	53	2.9×10^−3^
**Experiment 3**	GFP	103/1308		
	Hsc70 #1	29/1311	28	2.0×10^−5^
	Hsc70 #2	43/2056	27	2.7×10^−5^

Hela cells expressing B12-HA were seeded onto coverslips and transfected with siRNAs directed against GFP or Hsc70. Three days later, the coverslips were processed for anti-B12 immunofluorescence. The fraction of cells with DJANGOS was determined in at least 10 random fields for each condition. Three independent experiments were performed using a pool of siRNAs (experiment 1) or two different siRNAs directed against Hsc70 (experiments 2 and 3). Hsc70 siRNA reduced Hsc70 mRNA ∼50 fold.

The carboxy-terminal domain of B12 resides in the ER lumen and possesses a DUF1977 domain commonly found in DNAJ proteins. To test whether this segment of B12 is required for DJANGOS formation, we expressed a C-terminal truncation mutant of B12 (B12ΔC), which retains only 10 amino acids of the luminal domain. In contrast to the J-domain mutants, B12ΔC robustly induces DJANGOS formation (Figure 8B, right panel), demonstrating that the luminal portion of B12 is dispensable for this activity.

## Discussion

The maintenance of membrane identity is essential for cell function. Therefore, cells have evolved complex mechanisms to ensure the proper segregation of cellular components into specific membranes and the proper localization of membrane structures within the cell. We show here that over-expression of DNAJB12 or DNAJB14 causes the formation of elaborate membranous structures within the nucleus, which we name DJANGOS. B12 and B14 themselves are in DJANGOS, as are a variety of other membrane and luminal ER proteins and INM proteins. DJANGOS also contain Hsc70, a chaperone partner of B12 and B14, which is primarily cytoplasmic but can also localize to the nucleus and nuclear envelope [15], [24]. DJANGOS formation occurs in HeLa cervical carcinoma cells, primary human foreskin fibroblasts and monkey CV1 kidney cells, demonstrating that these structures are not restricted to a particular cell type or species.

Strikingly, all DJANGOS within a given nucleus are of a similar size and shape at the light microscope level, suggesting that these structures form coordinately. Indeed, live cell imaging revealed that DJANGOS form at the nuclear envelope within a few minutes of each other and grow in parallel. Remarkably, during mitosis all DJANGOS rapidly disappear immediately before nuclear division and synchronously reform in both daughter cells, similar to the nuclear envelope itself. Rarely, we observed B12 structures in the cytoplasm, which form in the nucleus and then escape into the cytoplasm either as membrane blebs or as discrete balloon-like bodies trailing a “string” of material back to the nucleus.

Electron microscopy of cells over-expressing B12 and B14 revealed the presence of a variety of membranous structures within the nuclear volume, ranging from small clusters of tubes spread around the nuclear periphery to massive, elaborate complexes of tubes, sheets, and multi-layered concentric whorls. In addition to these complex single-walled structures, cells over-expressing B12 or B14 contain double-walled membrane bodies near the nuclear periphery. All of the direct connections we observed between DJANGOS and the nuclear envelope involve these double-membrane bodies in association with atypical NPCs. Unlike normal NPCs, the NPC-like structures associated with DJANGOS were attached only to the ONM, rather than being anchored at the junction of the ONM and INM. In addition, the membrane attached to these NPCs is less highly curved than in normal NPCs, suggesting that the interaction of nucleoporins with the membrane is aberrant. In this configuration, both the INM and the ONM appear to flow around the entire circumference of the NPC into the interior of the nucleus. This implies that the NPC does not form its usual barrier between the INM and ONM, although it evidently preserves some barrier function because ribosomes and Sec61γ are not present in DJANGOS. The interior of these double-membrane bodies appears to represent a unique topological domain within the cell; it is internal to an attached NPC yet is separated from the nucleoplasm by a double-membrane. The contents of these bodies thus remain undefined.

DJANGOS contain both luminal and membrane ER markers. The ER lumen is continuous with the intramembrane space of the nuclear envelope, which in turn is connected to the interior of DJANGOS via the double-membrane body. These findings strongly suggest that DJANGOS are formed by invasion of the nucleus by the ER itself, rather than by *de novo* synthesis of membranes within the nucleus, which then somehow contain ER membrane proteins and enclose ER luminal contents. Furthermore, the ER, like DJANGOS, consists primarily of single-walled tubes and sheets, also suggesting that the ER and DJANGOS form via a related mechanism. However, atlastin 1, which is required to form the three-way junctions characteristic of the ER is absent from DJANGOS (unpublished results), perhaps explaining the predominance of single-walled, non-branching tubes within DJANGOS. Because the atypical NPCs appear to be the only point of contact between DJANGOS and cytoplasmic ER, we hypothesize that they are the conduit for membrane and protein ER components to enter the nuclear volume. The EM images shown in Figures 5A and B imply that the membrane of the double-membrane bodies that is topologically equivalent to INM spools off the prodigious amount of membrane necessary to generate the single-walled tubes and sheets that form the bulk of DJANGOS. Thus DJANGOS consist of three components: the atypical NPC attached to the outer membrane of the nuclear envelope, a connected double-walled membrane structure, and single-walled tubes and sheets that emerge from the double-walled structure and form the bulk of the DJANGOS.

Our genetic and biochemical analysis provides insight into the mechanism of DJANGOS formation. The J-domain of B12 and B14 is located on the cytoplasmic side of the ER membrane, where it normally engages Hsc70. Co-immunoprecipitation demonstrated stable complex formation between B12 and Hsc70 in cells containing DJANGOS. Mutation of the essential histidine in the J-domain disrupts the complex between B12 and Hsc70 and prevents DJANGOS formation, and Hsc70 knock-down inhibits DJANGOS formation. Taken together, these results strongly suggest that the normal chaperone function of the DNAJ/Hsc70 complex is essential for DJANGOS formation. The DNAJB12/Hsc70 interaction is also required for DNAJB12-mediated ERAD [17]–[19]. In contrast, the luminal domain of B12 is not required for DJANGOS formation.

We can envision two broad classes of models to explain the initiation of DJANGOS formation, depending on whether DJANGOS arise from the modification of existing NPCs or if the atypical NPCs form *de novo*. B12 or B14 over-expression may modify NPC function at a minority of pre-existing pores, allowing membranes to flow past the modified pore into the nucleus. Alternatively, elevated B12 or B14 and associated proteins in the ONM may self-associate to act like a “drawstring” to pull together distal segments of the nuclear envelope to form the double-membrane structure or may cause a local invagination of the nuclear envelope. DNAJ protein complexes or the membrane deformations they cause may then recruit NPC components to initiate atypical NPC formation at the neck and the invasion of membranes into the nucleus. The drawstring model can readily explain the existence of normal-appearing NPCs in the membranes of the double-membrane bodies inside the nucleus (*e.g.*, Figure 6A). We note that adjacent to the J-domain the cytoplasmic domain of B12 and B14 has a hydrophobic segment in addition to its membrane-spanning domain which might imbed into the ONM and drive membrane curvature, contributing to DJANGOS formation. The reticulons appear to play such a function in bending of the ER membrane and possibly the nuclear membranes at nuclear pores [25]–[28]. The membrane curving activity of reticulon 1, perhaps in cooperation with similar activity in B12 or B14, may induce membrane curvature at the nuclear pore or during the formation of the double-membrane structures or the highly-curved single-wall tubes that comprise the bulk of the DJANGOS. It is also possible that B12 and B14 affect the localization or function of reticulon 1 itself, influencing the topology of the resulting curvature. In addition, Hsc70 can localize to the NPC [26], [29], recruit proteins to the NPC [30], and participate in nuclear import and export of various proteins [15], [31]–[33]. Thus, elevated levels of B12 or B14 may modulate the NPC-associated activities of Hsc70, resulting in the initiation of DJANGOS formation.

Any model of DJANGOS formation must also be consistent with the spacing of DJANGOS beneath the entire circumference of the nuclear envelope and the observation that they can form in the intact nuclear envelope as well as in the envelope that forms immediately after mitosis. The regular spacing may result from the growth of DJANGOS from a subset of NPCs that is non-randomly distributed across the surface of the nucleus, hinting at a heretofore unknown level of nuclear organization. Alternatively, mechanical stresses or weaknesses in the nuclear envelope or the underlying lamina may preferentially drive the formation of atypical NPCs and DJANGOS at regular distances from each other.

In addition to the simple nucleoplasmic reticulum, complex intranuclear membrane structures have been observed in a variety of normal and cancerous human tissues. Structures similar to the bundled single-walled form of DJANGOS are present in hypertrophic cardiac muscle cells [34], alveolar epithelium [35], alveolar cell carcinomas [36], Novikoff hepatoma cells [37] and gastric carcinomas [38]. The Nucleolar Channel System (NCS) is a one-micron structure of single-walled membranous tubes embedded in an electron-dense matrix in the nuclei of postovulatory endometrial cells [39], [40]. The function of these structures is not known, and their biochemical composition is unexplored, other than the presence of some ER and INM components shared between NCS and DJANGOS. Expression of various exogenous proteins can also induce the appearance of membrane structures in the nucleus. Over-expression of the nucleolar protein Nopp140 created nuclear structures designated “R-rings,” that resembled the NCS [41], [42]. A single-walled membrane “stalk” connecting the R-rings to the nuclear envelope suggested that these structures formed from the INM. Intranuclear membranes, most commonly concentric multi-lamellar whorls, can also be induced by expression of a variety of other wild-type and mutant cellular, viral, or artificial proteins, including components of the nuclear envelope [43]–[46]. Thus, the formation of complex membranous structures in the nucleus can be instigated by many stimuli, ranging from the physiological or pathological events responsible for the naturally occurring structures to a variety of over-expressed proteins. It is not known if these inputs trigger a common mechanism that leads to the proliferation of membranes within the nucleus, or whether these structures arise through different mechanisms. None of the previously reported structures is known to undergo rapid, synchronous formation or to contain atypical NPCs, nor is it known if DNAJ or Hsc70 activity is required for their formation.

We did not observe DJANGOS unless B12 or B14 was over-expressed. Nevertheless, several findings suggest that formation of these structures is not merely a non-specific response to cell stress but rather an emergent property of B12 and B14 that results from their underlying biochemical activities. The regular distribution of DJANGOS that appear similar within single nuclei at the light microscope level, as well as their rapid and synchronous formation and disappearance, indicates that they are the result of a regulated process. This synchronous timing may reflect a transient signaling event, such as a modification of the DNAJ protein, Hsc70, or various NPC components. In addition, untagged B12 can induce formation of DJANGOS, and endogenous levels of B12 are required for DJANGOS formation when B14 is over-expressed. Most importantly, the co-chaperone activity of B12 and B14 is required for DJANGOS formation. The existence of naturally occurring intranuclear membrane complexes that resemble DJANGOS further suggests that these intriguing structures play a physiological or pathological role.

As well as participating in ERAD and DJANGOS formation, B12 and B14 are required for SV40 to exit the ER [14]. Hsc70 is also involved in nuclear entry of the human papillomavirus L2 capsid protein and adenoviral DNA [47], [48]. It is possible that endogenous B12 and B14 form small DJANGOS that have eluded detection but provide a conduit to deposit incoming viral genomes (or other molecules) inside the nucleus. Further studies of these unusual structures will provide insight into the complex processes that ensure that cellular structures maintain their structural and biochemical identity. Furthermore, such studies are likely to elucidate novel aspects of NPC formation and function, and may also illuminate new features of virus infection.

## Materials and Methods

### Cells and Reagents

CV1 and 293T cells were purchased from the American Type Culture Collection. HeLa/Sen2 cells, a clonal line highly susceptible to SV40 infection [22], are referred to as HeLa cells. HeLaM cells were obtained from W. Mothes (Yale University). Primary human foreskin fibroblasts were obtained from the Yale Skin Diseases Research Center. All cells were cultured in DMEM medium containing 10% fetal bovine serum (FBS), 10 mM HEPES [pH 7.2] and antibiotics. FuGENE HD/6 transfection reagents were purchased from Promega and Lipofectamine RNAiMAX from Invitrogen. Protein A/G Plus agarose beads were purchased from Santa Cruz Biotechnology. The crosslinking agent, DSP (dithiobis[succinimidylpropionate]), was purchased from Pierce. Oligonucleotides were obtained from Integrated DNA Technologies (Coralville, IA). siRNA duplexes were purchased from Thermo Scientific (Waltham, MA).

Human DNAJB12 and DNAJB14 genes were purchased from Origene (Rockville, MD) and a DNAJB12 cDNA linked to turboGFP was purchased from Evrogen (Moscow, Russia). Standard techniques were used to create mutations or add an HA or fluorescent protein tag to these genes, which were inserted into the pBabe vector (Cell Biolabs, Inc, San Diego, CA) co-expressing the blasticidin resistance gene and packaged as retrovirus in 293T cells. The lamin B receptor-eGFP was obtained from W. Mothes. shRNAs were designed using the Invitrogen BLOCK-iT RNA interference (RNAi) designer, inserted into the pSiren vector (Clontech, Mountain View, CA) expressing the puromycin resistance gene, and packaged as retroviruses in 293T cells. Stable polyclonal cell lines expressing shRNA, DNAJB12 or DNAJB14 were created by infecting HeLa cells with the appropriate retroviral stock followed by selection with puromycin or blasticidin.

### Antibodies

Anti-HA antibodies were used for immunofluorescence (3F10 rat monoclonal, Roche Applied Science) or immunoblotting (rabbit sc-805, Santa Cruz Biotechnology). Other antibodies from Santa Cruz Biotechnology included emerin (sc-15378), lamin A (sc-6215), lamin B (sc-6216), PDI (sc-20132), RTN1/2 (sc-23881), RTN2 (sc-16682), RTN3 (sc-33599), and RTN4 (sc-25660). Anti-turboGFP was purchased from Evrogen, anti-BiP and anti-calnexin from Abcam, anti-nuclear pore Mab(414) from Covance, and anti-Hsc70 from Enzo (SPA-757). The Sec61γ antibody was the gift of P. Lusk (Yale University). Fluorescently-labeled donkey anti-mouse, rabbit and goat immunoglobulin G (IgG; H+L) were purchased from Invitrogen (Carlsbad, CA) and used as secondary antibodies. Horseradish peroxidase-labeled donkey anti-mouse, rabbit or goat secondary antibodies were purchased from Jackson Immunoresearch and used in immunoblots. A B12-specific monoclonal antibody (clone 2D15) was developed in collaboration with Ab-Mart (Arlington, MA) against the epitope PTDTTHATHR in human DNAJB12. This antibody performed well for immunofluorescence, immunoblotting, and immunoprecipitation. Immunoblotting with this antibody showed that B12 shRNA caused loss of the endogenous B12 protein and that exogenous B12 was expressed at higher levels than the endogenous protein (Figure S1C).

### Fluorescence Microscopy

Cells were plated in 24-well dishes containing 12 mm #1.5 circular coverslips and allowed to grow for at least three days. The cells were washed with phosphate-buffered saline (PBS), fixed for 15 min with 4% paraformaldehyde in PBS and washed four times with PBS. The cells were permeabilized for 20 min with 0.5% Triton X-100 in PBS followed by two PBS washes and one wash in PBS with 0.5% bovine serum albumin and 0.01% sodium azide (FSB). Cells were blocked with 5% normal donkey serum in FSB (Blocking Buffer) for one hour at room temperature, followed by two FSB washes and incubation overnight at 4°C in a humidified chamber with primary antibodies diluted in Blocking Buffer. After four washes with FSB, the samples were incubated at room temperature for one hour with a 1∶500 dilution of Alexa Fluor-conjugated secondary antibody supplemented with 300 nM DAPI (4′,6′-diamidino-2-phenylindole) in Blocking Buffer. The cells were washed three times in FSB, once in PBS and dipped three times in water before mounting in Prolong Gold Antifade (Invitrogen). Staining was visualized by using a Zeiss Axioskop microscope equipped with a x40 and x100 objective lens, a 1.6x optivar and a 10X eyepiece and fluorescence filters appropriate for DAPI and Alexa Fluor 488, 568/594 (Zeiss, Thornwood, NY). Cell images were captured using a QImaging camera and MetaMorph software using the same exposure settings for all samples in an experiment. Images were background subtracted using the sliding parabola method and color merged using the ImageJ program using the same intensity settings in cases where direct comparisons were made.

### Electron Microscopy

Cells were grown on coverslips, washed twice in PBS and fixed for 30 min in 4% paraformaldehyde and 2.5% gluteraldehyde in 0.1 M sodium cacodylate buffer [pH. 7.4] at room temperature followed by an additional 30 min at 4°C. The cells were washed 4x in cacodylate buffer, post-fixed in 1% osmium tetroxide, *en bloc* stained in 2% aqueous uranyl acetate for an hour, then rinsed and dehydrated in an ethanol series followed by epon resin (Embed812 EMS) infiltration and baking overnight at 60°C. 60 nm sections cut using a Leica UltraCut UCT were collected on formvar/carbon coated grids and contrast stained using 2% uranyl acetate and lead citrate. For tomography, 200 nm sections were contrast stained. For cryoimmuno-electron microscopy, cells were fixed in 4% paraformaldehyde/0.1% gluteraldehyde in PBS for 30 min followed by 4% PFA for one hour. Cells were rinsed in PBS, scraped and re-suspended in 10% gelatin and placed in 2.3 M sucrose overnight at 4°C. Cells were transferred to aluminum pins and frozen rapidly in liquid nitrogen. 65 nm thick sections were placed on carbon/formvar coated grids and floated in a dish of PBS for immunolabeling with 1∶25 anti-BiP. 10 nm Protein A gold (UtrechtUMC) was used as a detection reagent. Grids were rinsed in PBS, fixed using 1% gluteraldehyde for five min, rinsed, and transferred to a UA/methylcellulose drop before drying. Samples were viewed on a FEI Tecnai Biotwin TEM at 80 Kv, and images were taken using a Morada CCD camera and iTEM (Olympus) software. Tomography was performed on a FEI Tecnai TF20 FEG operated at 200 KV. Data was collected on a FEI Eagle 4kX4k CCD camera and reconstructed using Imod ++. 3D renderings were generated using Amira (FEI). ONM, INM and parts of the nuclear lamina were selected using a threshold-based tool (‘magic wand’), and NPCs were traced manually.

### shRNA knockdown combined with B12 or B14 overexpression

HeLa cells expressing shRNAs targeting B12 or B14 or an irrelevant gene (Lib1) were transduced with B12-HA or B14-HA as above. Anti-HA immunofluorescence was conducted as above and the fraction of cells with DJANGOS was determined for each condition by combining counts from multiple random fields. A 2-tailed F-test was used to determine that an unequal variance 2-tailed t-test was appropriate to identify significant changes.

### siRNA tranfection

HeLa cells expressing B12-HA were seeded on 12 mm circular coverslips in a 24-well plate. One day later, cells were transfected with siRNAs against Hsc70 or GFP using Lipofectamine RNAiMAX. After three days, the cells were fixed and stained for DNAJB12 immunofluorescence as above. The fraction of cells with DJANGOS was scored in at least 10 random fields for each condition. A 2-tailed F-test was used to determine that an equal variance 2-tailed t-test was appropriate to identify significant changes in the fraction of Hsc70 knock-down cells with DJANGOS compared to cells transfected with siGFP. Knock-down of Hsc70 was confirmed by qRT-PCR. Total cellular RNA was isolated using an RNeasy mini kit (Qiagen, Valencia, CA), including on-column DNase digestion to remove contaminating DNA. 1 µg RNA was converted to cDNA using an iScript cDNA synthesis kit (Bio-Rad, Hercules, CA), and the cDNA was subjected to quantitative reverse transcriptase real-time PCR (qRT-PCR) in triplicate using IQ SYBR green Supermix and a MyIQ single color RT-PCR cycler (both from Bio-Rad) with normalization to GAPDH.

### Live Cell Imaging

10^4^ cells were seeded on Lab-Tek 8 chambered coverslip (Nunc) previously treated with 0.1% Poly-L-Lysine (Sigma) for 10 minutes at room temperature. CV1 cells were transfected with 0.2 µg DNA each of lamin B receptor-eGFP and DNAJB12-mCherry plasmids per well with 1.2 µl/well FuGENE 6 reagent. HeLa and HeLaM cells were transfected with 0.25 µg DNA of each construct per well with 1.5 µl FuGENE HD reagent. 36 h post-transfection, live-cell imaging at 37°C was started with a 60x objective on a Volocity spinning-disc microscope (Perkin-Elmer). Movies were created by assembling still images taken every 15 minutes. Analysis and editing were performed on Volocity and ImageJ software.

### Immunoprecipitation and Blotting

HeLa cells were grown on 150 mm dishes for four days, washed twice with cold PBS and incubated with 1 mM DSP in PBS for two hours at 4°C. Cells were washed with cold PBS and the proteins extracted with RIPA buffer for 30 min at 4°C. Nuclei were pelleted and the supernatants used directly for immunoblotting or immunoprecipitated by incubating 1 µg of antibody per mg of total protein overnight at 4°C, followed by precipitation with protein A/G beads. After washing, crosslinks were reversed and the proteins liberated by heating at 95°C for 10 minutes in 2X Laemmli sample buffer with 200 mM DTT. After SDS polyacrylaminde gel electrophoresis, the proteins were transferred to Immobilon-P membranes (Millipore), blocked with 5% non-fat dry milk and incubated with primary antibodies overnight at 4°C. The blots were washed, exposed to horseradish peroxidase-labeled secondary antibody and developed using SuperSignal West Femto (Thermo).

## Supporting Information

Figure S1 **Immunofluorescence of DJANGOS.** **A.** Immunofluorescent staining of DJANGOS in HeLa cells over-expressing B14-HA. Fixed cells were stained with an anti-HA antibody (green), and the nuclei were counter-stained with DAPI (blue). **B.** HeLa cells were infected with retroviruses expressing B12-tGFP and B14-HA. After selection, fixed cells were stained with anti-tGFP to detect B12-tGFP (in green) and with anti-HA to detect B14-HA (in red). The same confocal slice is shown in both panels. **C.** RIPA extracts from variously engineered HeLa cells were subjected to immunoblotting and probed with the anti-B12 monoclonal antibody. Lane 1, HeLa cells expressing an shRNA directed against an irrelevant gene, showing endogenous B12. Lane 2, HeLa cells expressing B12-tGFP but no shRNA. Lane 3, HeLa cells expressing an shRNA directed against B12. **D.** HeLa and CV1 cells, as indicated, transduced with untagged human B12 were stained with anti-B12 monoclonal antibody. A single confocal slice is shown for both cell types.
(TIF)

Figure S2 **Co-localization of DJANGOS with reticulons and lamin B receptor and visualization of the nucleoplasmic reticulum.** Top row, HeLa cells over-expressing B12-tGFP were fixed and stained with anti-tGFP (green) and anti-RTN1/2 (red). Middle row, HeLa cells transfected with LBR-eGFP (green) and B12-mCherry (red) expression vectors. Bottom row, HeLa cells were stained for emerin (green) and B12 (red). The arrows indicate nucleoplasmic reticulum. Each row shows the same confocal slice. Areas of co-localization in all of the merged images appear yellow.
(TIF)

Figure S3 **Simple DJANGOS and nucleoplasmic reticulum visualized by electron microscopy.** **A.** HeLa cell over-expressing B12-HA and E2. The arrow points to a small bundle of single-walled nuclear tubes. **B. and C.** Unmodified HeLa cells were visualized by electron microscopy. Panel B shows a single nuclear intrusion representing the nucleoplasmic reticulum in longitudinal section; Panel C shows cross-sections of two nucleoplasmic reticulum intrusions, both of which contain nuclear pores (arrow) and lamin staining.
(TIF)

Movie S1 **Tomographic stacks and rendering.** A series of slices generated from EM tomography are shown as a movie progressing through the structure. The opening frame shows a cross-section of the double-membrane body (which also contains a vesicle) entering the nucleus at an atypical nuclear pore, with the cytoplasm at the top. The movie first shows the stack progressing through the cross-section. The movie then shows a perpendicular stack showing the pore at the neck of the structure end-on, followed by the rotation of the 3D rendering. The internal vesicle was removed from the rendering for clarity.
(MOV)

Movie S2 **Formation of DJANGOS in an interphase cell.** HeLa cells were transfected with plasmids encoding B12-mCherry and LBR-eGFP and subjected to live cell imaging as described in the methods, with images captured every 15 minutes.
(MOV)

Movie S3 **Behavior of DJANGOS during cell division.** HeLaM cells were handled as described in legend to Movie S2. This video is the source of the images shown in Figure 7A.
(MOV)

Movie S4 **Cytoplasmic billows.** CV1 cells were handled as described in legend to Movie S2. This video is the source of the image shown in Figure 7B, left panel.
(MOV)

Movie S5 **Balloon-like cytoplasmic extrusions.** CV1 cells were handled as described in legend to Movie S2. This video is the source of the image shown in Figure 7B, right panel.
(MOV)
