# Supplementary figures and images for: Expression of DNAJB12 or DNAJB14 Causes Coordinate Invasion of the Nucleus by Membranes Associated with a Novel Nuclear Pore Structure
Source: PLoS One. 2014 Apr 14;9(4):e94322. doi: 10.1371/journal.pone.0094322 (PMC3986390; doi:10.1371/journal.pone.0094322)

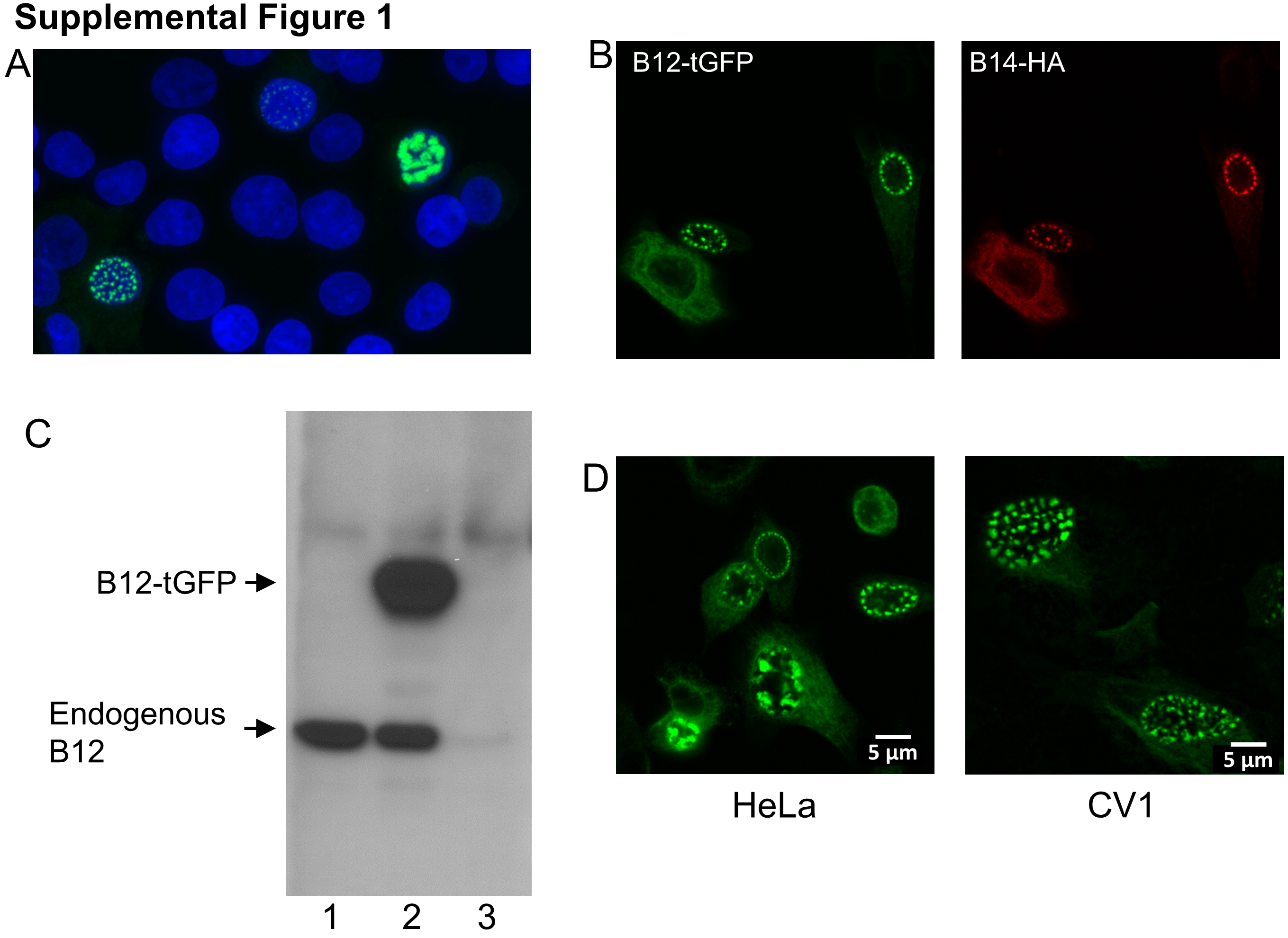

Supplement: Figure S1 — Immunofluorescence of DJANGOS. A. Immunofluorescent staining of DJANGOS in HeLa cells over-expressing B14-HA. Fixed cells were stained with an anti-HA antibody (green), and the nuclei were counter-stained with DAPI (blue). B. HeLa cells were infected with retroviruses expressing B12-tGFP and B14-HA. After selection, fixed cells were stained with anti-tGFP to detect B12-tGFP (in green) and with anti-HA to detect B14-HA (in red). The same confocal slice is shown in both panels. C. RIPA extracts from variously engineered HeLa cells were subjected to immunoblotting and probed with the anti-B12 monoclonal antibody. Lane 1, HeLa cells expressing an shRNA directed against an irrelevant gene, showing endogenous B12. Lane 2, HeLa cells expressing B12-tGFP but no shRNA. Lane 3, HeLa cells expressing an shRNA directed against B12. D. HeLa and CV1 cells, as indicated, transduced with untagged human B12 were stained with anti-B12 monoclonal antibody. A single confocal slice is shown for both cell types. (TIF) [file pone.0094322.s001.tif]

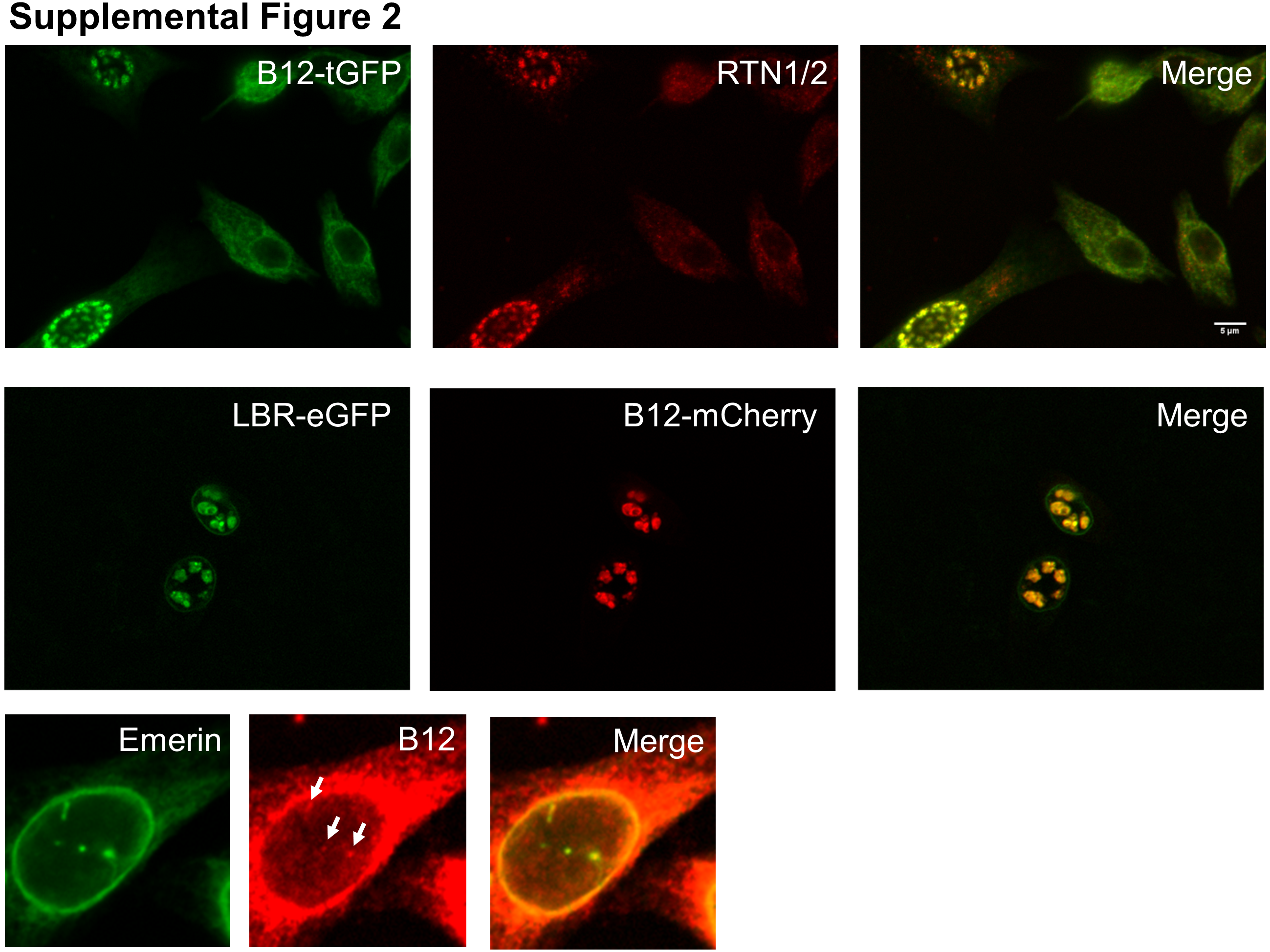

Supplement: Figure S2 — Co-localization of DJANGOS with reticulons and lamin B receptor and visualization of the nucleoplasmic reticulum. Top row, HeLa cells over-expressing B12-tGFP were fixed and stained with anti-tGFP (green) and anti-RTN1/2 (red). Middle row, HeLa cells transfected with LBR-eGFP (green) and B12-mCherry (red) expression vectors. Bottom row, HeLa cells were stained for emerin (green) and B12 (red). The arrows indicate nucleoplasmic reticulum. Each row shows the same confocal slice. Areas of co-localization in all of the merged images appear yellow. (TIF) [file pone.0094322.s002.tif]

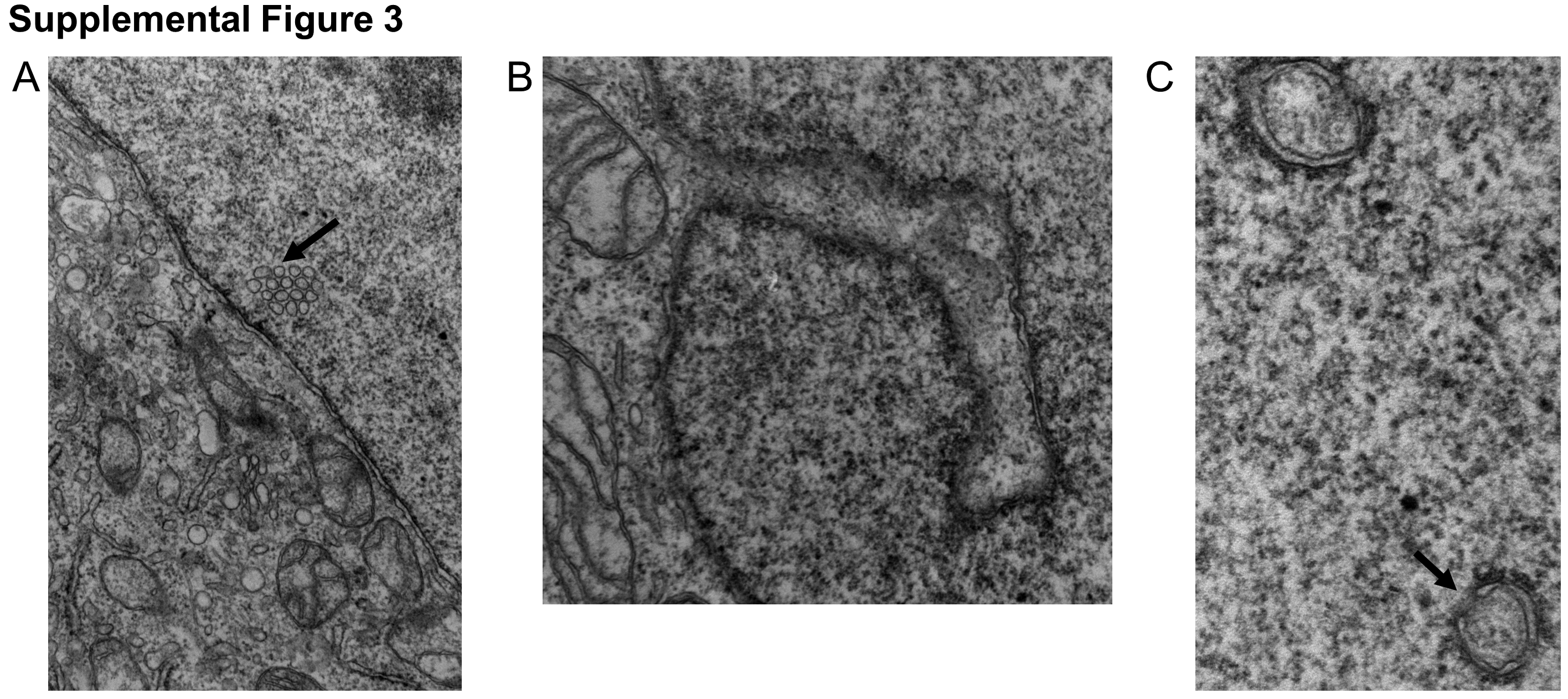

Supplement: Figure S3 — Simple DJANGOS and nucleoplasmic reticulum visualized by electron microscopy. A. HeLa cell over-expressing B12-HA and E2. The arrow points to a small bundle of single-walled nuclear tubes. B. and C. Unmodified HeLa cells were visualized by electron microscopy. Panel B shows a single nuclear intrusion representing the nucleoplasmic reticulum in longitudinal section; Panel C shows cross-sections of two nucleoplasmic reticulum intrusions, both of which contain nuclear pores (arrow) and lamin staining. (TIF) [file pone.0094322.s003.tif]
